# **Ventilation strategies in cardiogenic shock: insights from the** FRENSHOCK **observational registry**

**DOI:** 10.1007/s00392-024-02551-x

**Published:** 2024-10-23

**Authors:** Kim Volle, Hamid Merdji, Vincent Bataille, Nicolas Lamblin, François Roubille, Bruno Levy, Sebastien Champion, Pascal Lim, Francis Schneider, Vincent Labbe, Hadi Khachab, Jeremy Bourenne, Marie-France Seronde, Guillaume Schurtz, Brahim Harbaoui, Gerald Vanzetto, Charlotte Quentin, Nicolas Combaret, Benjamin Marchandot, Benoit Lattuca, Caroline Biendel, Guillaume Leurent, Laurent Bonello, Edouard Gerbaud, Etienne Puymirat, Eric Bonnefoy, Nadia Aissaoui, Clément Delmas, FRENSHOCK Investigator, N Aissaoui, F Bagate, M Beuzelin, C Biendel, F Boissier, L Bonello, E Bonnefoy-Cudraz, M Boughenou, S Boule, J Bourenne, N Brechot, C Bruel, A Cariou, P Castellant, S Champion, K Chaoui, M Chatot, N Combaret, N Debry, X Delabranche, C Delmas, J Dib, R Favory, E Filippi, R Gallet, F Ganster, P Gaudard, E Gerbaud, B Harbaoui, P Henry, B Herce, F Ivanes, J Joffre, P Karoubi, H Khachab, K Khalif, K Klouche, V Labbe, M Laine, N Lamblin, B Lattuca, Y Lefetz, G Lemesle, P Letocart, G Leurent, B Levy, G Louis, J Maizel, J Mansourati, S Manzo-Silberman, S Marchand, B Marchandot, S Marliere, J Mootien, F Mouquet, L Niquet, A Paternot, V Probst, E Puymirat, C Quentin, G Range, N Redjimi, J Richard, F Roubille, C Saint Etienne, F Schneider, G Schurtz, M Seronde, J Ternacle, G Vanzetto, E Zogheib

**Affiliations:** 1 Intensive Cardiac Care Unit, Cardiology Department, Rangueil University Hospital, 31059 Toulouse, France; 2 Faculté de Médecine, Medical Intensive Care Unit, Université de Strasbourg (UNISTRA), Strasbourg University Hospital, Nouvel Hôpital Civil, Strasbourg, France; 3 Association pour la diffusion de la médecine de prévention (ADIMEP)–INSERM UMR1295 CERPOP –Toulouse Rangueil University Hospital (CHU), Toulouse, France; 4 Urgences Et Soins Intensifs de Cardiologie, CHU Lille, University of Lille, Inserm U1167, 59000 Lille, France; 5 PhyMedExp, Cardiology Department, Université de Montpellier, INSERM, CNRS, INI-CRT, CHU de Montpellier, France; 6 CHRU Nancy, Réanimation Médicale Brabois, Vandoeuvre-Les Nancy, France; 7 Clinique de Parly 2, Ramsay Générale de Santé, 21 Rue Moxouris, 78150 Le Chesnay, France; 8 Service de Cardiologie, Univ Paris Est Créteil, INSERM, IMRB, AP-HP, Hôpital Universitaire Henri-Mondor, F-94010 Créteil, France; 9 Médecine Intensive-Réanimation, Hôpital de Hautepierre, Hôpitaux Universitaires de Strasbourg, Strasbourg, France; 10 Medical Intensive Care Unit, Tenon Hospital, Assistance Publique- Hôpitaux de Paris, Paris, France; 11 Intensive Cardiac Care Unit, Department of Cardiology, CH d’Aix en Provence, Avenue Des Tamaris 13616, cedex 1 Aix-en-Provence, France; 12 Service de Réanimation Des Urgences, Aix Marseille Université, CHU La Timone 2, Marseille, France; 13 Service de Cardiologie CHU Besançon, Marseille, France; 14 Cardiology Department, Hôpital Croix-Rousse and Hôpital Lyon Sud, Hospices Civils de Lyon, University of Lyon, CREATISUMR 5220INSERM U1044INSA-15, Lyon, France; 15 Department of Cardiology, Hôpital de Grenoble, 38700 La Tronche, France; 16 Service de Reanimation Polyvalente, Centre Hospitalier Broussais St Malo, 1 Rue de La Marne, 35400 St Malo, France; 17 Department of Cardiology, CHU Clermont-Ferrand, CNRS, Université Clermont Auvergne, Clermont-Ferrand, France; 18 Université de Strasbourg, Pôle d’Activité Médico-Chirurgicale Cardio-Vasculaire, Nouvel Hôpital Civil, Centre Hospitalier Universitaire, 67091 Strasbourg, France; 19 Department of Cardiology, Nîmes University Hospital, Montpellier University, Nîmes, France; 20 Department of Cardiology, CHU Rennes, Inserm, LTSI—UMR 1099, Univ Rennes 1, 35000 Rennes, France; 21 Intensive Care Unit, Department of Cardiology, Aix-Marseille UniversitéAssistance Publique-Hôpitaux de Marseille, Hôpital NordMediterranean Association for Research and Studies in Cardiology (MARS Cardio), F-13385 Marseille, France; 22 Intensive Cardiac Care Unit and Interventional Cardiology, Hôpital Cardiologique du Haut Lévêque, 5 Avenue de Magellan, 33604 Pessac, France; 23 Bordeaux Cardio, Thoracic Research Centre, U1045, Bordeaux University, Hôpital Xavier Arnozan, Avenue du Haut Lévêque, 33600 Pessac, France; 24 Department of Cardiology, Assistance Publique-Hôpitaux de Paris (AP-HP), Hôpital Européen Georges Pompidou, 75015 Paris, France; 25 Université de Paris, 75006 Paris, France; 26 Intensive Cardiac Care Unit, Lyon Brom University Hospital, Lyon, France; 27 Medical Intensive Care Unit, Cochin Hospital, Assistance Publique-Hôpitaux de Paris, Centre–Université de Paris, Medical School, Paris, France; 28 Institute of Metabolic and Cardiovascular Diseases (I2MC), UMR-1048, National Institute of Health and Medical Research (INSERM), Toulouse, France; 29 Recherche Et Enseignement en Insuffisance Cardiaque Avancée Assistance Et Transplantation (REICATRA), Institut Saint Jacques, CHU Toulouse, France; 30 Université Paul Sabatier, Toulouse III, Toulouse, France

**Keywords:** Cardiogenic shock, Prognosis, Mechanical ventilation, Non-invasive ventilation, Mortality

## Abstract

**Background:**

Despite scarce data, invasive mechanical ventilation (MV) is widely suggested as first-line ventilatory support in cardiogenic shock (CS) patients. We assessed the real-life use of different ventilation strategies in CS and their influence on short and mid-term prognosis.

**Methods:**

FRENSHOCK was a prospective registry including 772 CS patients from 49 centers in France. Patients were categorized into three groups according to the ventilatory supports during hospitalization: no mechanical ventilation group (NV), non-invasive ventilation alone group (NIV), and invasive mechanical ventilation group (MV). We compared clinical characteristics, management, and occurrence of death and major adverse event (MAE) (death, heart transplantation or ventricular assist device) at 30 days and 1 year between the three groups.

**Results:**

Seven hundred sixty-eight patients were included in this analysis. Mean age was 66 years and 71% were men. Among them, 359 did not receive any ventilatory support (46.7%), 118 only NIV (15.4%), and 291 MV (37.9%). MV patients presented more severe CS with more skin mottling, higher lactate levels, and higher use of vasoactive drugs and mechanical circulatory support. MV was associated with higher mortality and MAE at 30 days (HR 1.41 [1.05–1.90] and 1.52 [1.16–1.99] vs NV). No difference in mortality (HR 0.79 [0.49–1.26]) or MAE (HR 0.83 [0.54–1.27]) was found between NIV patients and NV patients. Similar results were found at 1-year follow-up.

**Conclusions:**

Our study suggests that using NIV is safe in selected patients with less profound CS and no other MV indication.

NCT02703038

**Graphical abstract:**

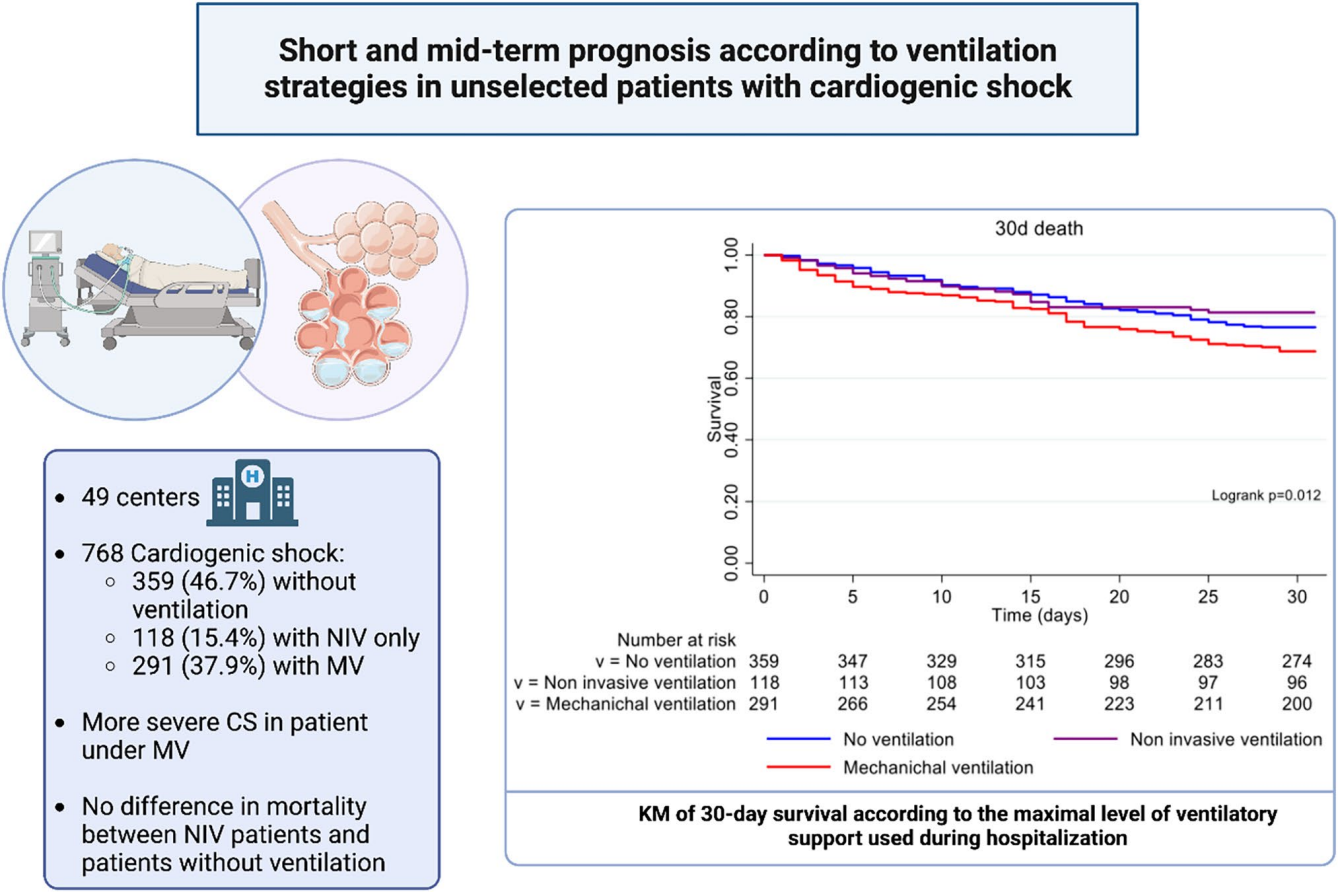

**Supplementary Information:**

The online version contains supplementary material available at 10.1007/s00392-024-02551-x.

## Introduction

Cardiogenic shock (CS) is consensually considered as a primary cardiac dysfunction with low cardiac output leading to critical end-organ hypoperfusion [1, 2]. The most frequent causes of CS are non-ischemic cardiomyopathy and myocardial infarction [3–5]. Despite recent therapeutic advances in medication and intervention, the short-term mortality of CS remains high between 30 and 50% [5–7].

CS patients usually present an increase in pulmonary capillary pressures responsible for an alteration in gas exchange revealed by acute pulmonary oedema (APE) and respiratory distress (57%) requiring oxygen support and, for the most severe (43%), mechanical ventilation (MV)[8]. These ventilatory therapies, such as non-invasive ventilation (NIV) and MV have been increasingly used in recent years, although no prospective trial has been conducted to date in CS [8]. Most recommendations propose MV as first-line ventilatory support in CS but are based on a low level of evidence (1C), i.e., expert consensus [1, 2, 9–13]. Nevertheless, the use of MV is frequently associated with excess mortality even in CS patients and its prolonged use is associated with increased length of stay, increased morbidity and mortality, and significant loss of autonomy in case of survival [14]. NIV is recommended as first-line treatment for APE with hypoxia (SpO2 < 90%) with a class IIa grade [13] since it reduces the need for intubation and early mortality compared to traditional oxygen therapy. According to the Franck –Starling law, the effects of MV in CS may be difficult to predict. On one hand, the pathophysiology effects of NIV may be beneficial in CS patients with decreased LVEF as it may increase cardiac output. On the other hand, when there is isolated right ventricular (RV) dysfunction, positive pressure may be detrimental as the increase in RV afterload may precipitate or aggravate RV failure, explaining why NIV was contraindicated for a long time in CS. Besides, CS patients may have encephalopathy leading to difficulties to conduct NIV [15–17].

Several registries provide conflicting results regarding the effect of NIV in the management of CS patients. For some, NIV is associated with an increased risk of complications and mortality probably due to delayed orotracheal intubation, whereas for others it seems to be effective and safe in this indication [8, 14, 18]. In all cases, the delay in the initiation of adapted ventilatory support seems to be associated with an over risk of mortality [19].

Based on the largest European prospective cohort of unselected CS to date, we aimed to assess characteristics and outcomes of CS according to the type of ventilatory support used. Our secondary objective was to determine prognostic factors of the need for MV in CS patients.

## Methods

### Patient population

FRENSHOCK is a prospective multicenter observational registry conducted in metropolitan France for 6 months between April and October 2016 in intensive care unit (ICU) and intensive cardiac care units (ICCU) (NCT02703038). The methods used for this registry have been previously described [5, 20]. Briefly, the primary objective was to evaluate CS patients’ characteristics, management, and outcomes, with a new modified definition of CS as seen in routine clinical practice, on a nationwide scale.

All adult patients (≥ 18 years old) with CS were prospectively included in this registry if they met at least one criterion of each of the following three components: (1) hemodynamic criteria, defined as low systolic arterial pressure (SAP) < 90 mmHg and/or the need for maintenance with vasopressors/inotropes and/or a low CI < 2.2 L/min/m^2^; (2) left and/or right-heart overload, defined by clinical, radiology, blood tests, echocardiography, or invasive hemodynamics’ signs; and (3) signs of organ malperfusion, which could be clinical and/or biologic. Patients admitted after cardiopulmonary resuscitation were included if they fulfilled previously defined CS criteria. Patients could be included regardless of CS etiology, and whether CS was primary or secondary. Exclusion criteria were refusal or inability to consent. A diagnosis of CS was refuted in favor of alternative diagnoses, such as septic shock, refractory cardiac arrest, and post-cardiotomy CS [5, 20].

All institutions were invited to participate in the study, including university teaching hospitals, general and regional hospitals, as well as public and private hospitals that manage CS patients (ICCUs, surgical ICUs, medical ICUs, and general ICUs).

The study was conducted in accordance with the guidelines for good clinical practice and French law. Written consent was obtained for all the patients. The data recorded and their handling and storage were reviewed and approved by the CCTIRS (French Health Research Data Processing Advisory Committee) (n° 15.897) and the CNIL (French Data Protection Agency) (n° DR-2016–109).

### Data collection

Data on baseline characteristics, including demographics (age, gender, body mass index, social status), risk factors (hypertension, diabetes, current smoking, hypercholesterolemia, family history of coronary artery disease), and medical history [cardiomyopathy, myocardial infarction (MI), stroke, peripheral artery disease, chronic kidney disease, active cancer, chronic obstructive lung disease], were collected as previously mentioned. Clinical, biologic, and echocardiographic data were collected within the first 24 h after admission. Up to three CS triggers were determined for each patient by the local investigator, that is, ischemic (Type 1 or Type-2 acute myocardial infarction according to European guidelines); ventricular and supraventricular arrhythmia; conduction disorder; infectious disease; non-compliance (poor compliance with medical treatment or hygiene and diet rules, for example, stopping or skipping an angiotensin-converting enzyme inhibitor or beta-blocker treatment, deviation from a low sodium diet, etc.); or iatrogenesis. Investigators could also note other existing factors or etiologies. Such triggering factors were indicated as ‘other’. Information regarding the use of cardiac procedures, that is, coronary angiography and/or percutaneous coronary intervention (PCI); right-heart catheterization; the need for medications (inotropes, vasopressors, diuretics, and fibrinolysis) and organ replacement therapies such as MV (invasive or non-invasive); temporary mechanical circulatory support [intra-aortic balloon pump (IABP); venoarterial-extracorporeal membrane oxygenation (VA-ECMO) or Impella^®^ (Abiomed, Danvers, MA, USA)]; and renal replacement therapy (RRT) (continuous or intermittent) were collected. In-hospital complications were noted, such as stroke, bleeding and transfusions, hemolysis, thrombocytopenia, nosocomial infections, vascular complications, and death. Information on mortality was obtained directly by the local investigators (cause and date) through a 30-day and a 1-year follow-up.

### Statistical analysis

Continuous variables were reported as means (SD) or medians and interquartile ranges when appropriate. Discrete variables were described in numbers and percentages. Patients were categorized into three groups according to the maximal ventilatory supports used during hospitalization: no mechanical ventilation group (NV), non-invasive ventilation alone group (NIV), and invasive mechanical ventilation group (MV). Thus, patients who required invasive ventilation after NIV were classified into the MV group. We compared clinical characteristics, management, and occurrence of death and major adverse event (MAE) (death, heart transplantation or ventricular assist device) at 30 days and 1 year between the three groups. Differences between groups were tested using analyses of variance or Mann–Whitney non-parametric tests for continuous variables and using *χ*^2^ or Fisher’s exact tests for categorical variables. Factors independently associated with the use of MV were studied using multiple logistic regression. Survival analyses were conducted using the Kaplan–Meier method. Statistical analyses were performed using Stata *(Stata Statistical Software SE/17.0. StataCorp LLC. College Station. TX. USA.).* For all analyses, two-sided *p* values < 0.05 were considered significant.

## Results

### Study population

A total of 768 CS patients were included in 49 centers, among whom 359 (46.7%) did not require ventilation, 118 (15.4%) required NIV, and 291 (37.9%) MV. Clinical characteristics of these patients are presented in Table 1. The mean age, gender, and risk factors were similar in the three groups. There was no difference regarding medical history except less history of previous heart disease in the MV group (47.8 vs 50 in NIV and 64.9% in NV groups, *p* < 0.001). MV patients were less frequently under long-term cardiological treatments than those under NIV or NV (Beta-blockers, ACE inhibitors or ARB2, Furosemide, and anti-aldosterone).

**Table 1:** Clinical characteristics at admission according to the maximal level of ventilatory support used during hospitalisation

	No ventilation (*n* = 359)		Non-invasive ventilation (*n* = 118)		Mechanical ventilation (*n* = 291)		*p*
Male gender	260	72.4	75	63.6	214	73.5	0.111
Age (years), mean ± SD	68.0	± 13.5	69.1	± 16.1	61.7	± 15.0	0.963
BMI (kg/m^2^), mean ± SD	25.3	± 5.5	26.2	± 6.1	26.4	± 5.4	0.062
*n*	*340*		*118*		*283*		
Risk factors, *n* (%)							
Current smoker	91/351	25.9	32/116	27.6	83/269	30.9	0.397
Diabetes mellitus	110/358	30.7	35/118	29.7	71/291	24.4	0.189
Arterial hypertension	170	47.4	62	52.5	131	45.0	0.385
Dyslipidaemia	121	33.7	52	44.1	103	35.4	0.122
Medical history, *n* (%)							
History of cardiac disease	233	64.9	59	50.0	139	47.8	< 0.001
Ischaemic	123	34.3	35	29.7	71	24.4	0.024
Hypertrophic	8	2.2	2	1.7	1	0.3	0.092
Idiopathic	48	13.4	8	6.8	22	7.6	0.021
Toxic	26	7.2	3	2.5	5	1.7	0.002
Multisite pacing	34/358	9.5	12/118	10.2	17/291	5.8	0.169
Defibrillator	76/358	21.2	19/118	16.1	31/291	10.7	0.041
CABG	30/358	8.4	11/118	9.3	20/291	6.9	0.652
PCI	92/358	25.7	26/118	22.0	48/291	16.5	0.018
Peripheral artery disease	43/358	12.0	16/118	13.6	32/291	11.0	0.763
Ischemic stroke	29/358	8.1	6/118	5.1	25/291	8.6	0.472
Chronic renal failure	94/358	26.3	28/118	23.7	41/291	14.1	0.568
COPD	25/358	7.0	7/118	5.9	18/291	6.2	0.884
Active neoplasy	23/358	6.4	5/118	4.2	23/291	7.9	0.392
Previous medications, *n* (%)							
Aspirin	126	35.1	50	42.4	111	38.1	0.345
P2Y12 inhibitor	56	15.6	22	18.6	48	16.5	0.740
Statins	138	38.4	53	44.9	94	32.3	0.044
Betablockers	165	46.0	57	48.3	92	31.6	< 0.001
Vitamin K antagonist	98	27.3	26	22.0	40	13.8	0.361
Direct oral anticoagulant	29	8.1	13	11.0	14	4.8	0.067
ACE inhibitors or ARB	147	41.0	57	48.3	86	29.6	< 0.001
Sacubitril/valsartan	12	3.5	2	1.8	4	1.5	0.265
Furosemide	213	59.3	66	55.9	96	33.0	< 0.001
Aldosterone antagonist	73	20.3	11	9.3	24	8.3	< 0.001
Amiodarone	65	18.9	20	17.1	46	15.9	0.613
Proton pump inhibitor	144	40.8	43	36.8	88	30.7	0.030
Triggers							
Ischaemic	104	29.0	54	45.8	131	45.0	< 0.001
Mechanical	12	3.3	3	2.5	8	2.8	0.958
Ventricular arrhythmia	35	9.8	14	11.9	49	16.8	0.025
Atrial arrhythmia	62	17.3	13	11.0	33	11.3	0.057
Conductive disorders	7	2.0	2	1.7	9	3.1	0.653
Infectious	32	8.9	13	11.0	50	17.2	0.006
Non compliance	22	6.1	5	4.2	3	10	0.002
Iatrogenic	41	11.4	4	3.4	15	5.2	0.002
Other	46	12.8	11	9.3	44	15.1	0.281
None/undefined	73	20.3	15	12.7	22	7.6	< 0.001

*ACE* angiotensin-converting enzyme, *ARB* angiotensin-receptor blocker, *BMI* body mass index, *CABG* coronary artery bypass graft, *COPD* chronic obstructive pulmonary disease, *PCI* percutaneous coronary intervention, *SD* standard deviation

The most frequent CS triggers were ischemia, ventricular, and supraventricular arrhythmia without between groups difference except for ischemia, which was less frequent in NV group (29% vs 45.8 and 45%, *p* < 0.001).

The clinical, echography, and biologic presentations are presented in Table 2. Patients in the MV group were more often hospitalized in the ICU versus ICCU than patients under NIV or NV (58.8% vs 15.5 and 10.9% respectively, *p* < 0.001). MV patients presented with more previous cardiac arrest (21.3 vs 6.8 and 2.2%, *p* < 0.001), more skin mottling (51.4 vs 34.2 and 29.9%, *p* < 0.001), and higher lactate at admission than NIV or NV group (3.8 vs 2.7 vs 2.3 mmol/L, *p* < 0.001). Renal and hepatic functions were similar between groups. There were no between groups difference regarding echocardiography at admission besides tricuspid annular peak systolic velocity tissue doppler imaging which was higher in the MV group than NIV or NV (10 vs 8 vs 7 cm/s, *p* < 0.001).

**Table 2:** Clinical, echography, and biologic presentation according to the maximal level of ventilatory support used during hospitalization

	No ventilation (*n* = 359)		Non-invasive ventilation (*n* = 118)		Mechanical ventilation (*n* = 291)		*p*
Admission unit, *n* (%)							< 0.001
ICCU	236	89.1	87	84.5	91	41.2	
ICU	29	10.9	16	15.5	130	58.8	
Clinical presentation at admission							
Heart rate (bpm), mean ± SD	93	± 28	96	± 32	99	± 30	0.041
*n*	*358*		*118*		*290*		
SBP (mmHg), mean ± SD	100	± 23	103	± 24	102	± 28	0.439
*n*	*358*		*118*		*291*		
DBP (mmHg), mean ± SD	64	± 17	65	± 18	62	± 18	0.103
*n*	*358*		*118*		*290*		
Sinus rhythm, *n* (%)	177/356	49.7	60/118	50.9	159/291	54.6	0.449
Mottling, *n* (%)	88/294	29.9	39/114	34.2	128/249	51.4	< 0.001
Cardiac arrest, *n* (%)	8	2.2	8	6.8	62	21.3	< 0.001
Blood tests at admission							
Sodium (mmol/l), mean ± SD	134	± 6	136	± 5	135	± 6	0.003
*n*	*351*		*118*		*289*		
eGFR (mL/min/1.73 m^2^), mean ± SD	47.1	± 26.3	50.4	± 23.9	52.4	± 27.9	0.041
*n*	*346*		*117*		*286*		
Bilirubin (mg/L), median (IQR)	21 (11 – 32)		18 (11 – 31)		13 (8 – 22)		< 0.001
*n*	*250*		*81*		*211*		
Hemoglobin (g/dL), mean ± SD	12.6	± 2.3	12.6	± 2.3	12.4	± 2.5	0.741
*n*	*347*		*118*		*287*		
Arterial blood lactates (mmol/l), median (IQR)	2.3 (1.7–3.7)		2.7 (2.0 – 4.0)		3.8 (2.0 – 6.0)		< 0.001
*n*	*291*		*109*		*282*		
ASAT (IU/L), median (IQR)	89 (397–342)		54 (29 – 123)		103 (44 – 291)		0.022
*n*	*271*		*60*		*214*		
ALAT (IU/L), median (IQR)	61 (26–236)		38 (21 – 89)		62 (30 – 171)		0.014
*n*	*276*		*62*		*219*		
Nt proBNP (pg/mL), median (IQR)	12,711 (5003–30,289)		7708 (3659–13,352)		6541 (3466–13,700)		0.012
*n*	*116*		*22*		*85*		
BNP (pg/mL), median (IQR)	1437 (646–3274)		1193 (477–2436)		882 (271 – 2090)		0.010
*n*	*119*		*66*		*79*		
CRP (mg/L), median (IQR)	27 (10–55)		29 (8 – 60)		37 (8 – 107)		0.333
*n*	*219*		*54*		*133*		
Baseline echography							
LVEF (%), mean ± SD	25.8	± 13.6	27.8	± 12.7	26.4	± 13.3	0.398
*n*	*354*		*117*		*289*		
TAPSE (mm), mean ± SD	12.7	± 4.8	13.7	± 4.3	14.5	± 5.2	0.019
*n*	*142*		*21*		*96*		
PSVtdi (cm/s), median (IQR)	7 (6 – 9)		8 (7–10)		10 (7–13)		< 0.001
*n*	*101*		*25*		*80*		
Severe mitral regurgitation, *n* (%)	66/337	19.6	14/114	12.3	27/279	9.7	0.002
Severe aortic stenosis, *n* (%)	15/354	4.2	8/118	6.8	13/284	4.6	0.523
Severe aortic regurgitation, *n* (%)	5/351	1.4	0/116	0.0	5/285	1.8	0.479

*ALAT* alanine aminotransferase, *ASAT* aspartate aminotransferase, *CRP* C-Reactive Protein, *DBP* Diastolic Blood Pressure, *ICU* intensive care unit, *ICCU* intensive cardiac care unit, *IQR* interquartile range, *LVEF* left ventricular ejection fraction, *PSVtdi* peak systolic velocity tissue doppler imaging, *SBP* systolic blood pressure, *SD* standard deviation, *TAPSE* tricuspid annular plane systolic excursion

### In-hospital management

In-hospital management and parameters at discharge are presented in Table 3. The MV group received more volume expansion during the first 24 h of management than the NIV or NV groups (56.7 vs 49.2 vs 27.5%, *p* < 0.01). During hospitalization, they benefited from higher doses of inotropes and vasopressors (dobutamine, norepinephrine, and epinephrine). Moreover, organs support was more frequently used in this group with higher use of acute mechanical circulatory support (MCS) (36.8 vs 8.5 vs 6.4%, *p* < 0.01) mainly by VA-ECMO support (71% in the MV group), and higher use of RRT in the VM group (27.2 vs 8.5 vs 8.4% *p* < 0.01). On the contrary, there was a more common use of diuretics (89.4% vs 74.6% in the MV group, *p* < 0.01) and less acute MCS use (6.4%) from different types with a predominance of IABP (65.2%) in the NV group.

**Table 3:** In-hospital management and outcomes according to the maximal level of ventilatory support used during hospitalization

	No ventilation(*n*=359)		Non-invasive ventilation (*n*=118)		Mechanical ventilation(*n*=291)		*p*
Medications used, *n* (%)							
Diuretics	321	89.4	95	80.5	217	74.6	< 0.001
Volume expander	98	27.4	58	49.2	165	56.7	< 0.001
Dobutamine	307	85.5	87	73.7	238	81.8	0.014
If yes, maximum dose (g/kg/min):							< 0.001
5–10	215	70.0	65	74.7	125	52.5	
10–15	54	17.6	16	18.4	66	27.7	
>15	16	5.2	2	2.3	29	12.2	
Unknown	22	7.2	4	4.6	18	7.6	
Norepinephrine	137	38.2	34	28.8	239	82.1	< 0.001
If yes, maximum dose (mg/h):							< 0.001
< 1	39	28.5	12	35.3	35	14.6	
1–5	70	51.1	13	38.2	132	55.2	
>5	12	8.8	5	14.7	58	24.3	
Unknown	16	11.7	4	11.8	14	5.9	
Epinephrine	12	3.3	6	5.1	77	26.5	0.001
If yes, maximum dose (mg/h):							0.684
< 1	5	41.7	2	33.3	27	35.1	
1*–*5	4	33.3	4	66.7	32	41.6	
>5	1	8.3	0	0.0	13	16.9	
Unknown	2	16.7	0	0.0	5	6.5	
Norepinephrine + dobutamine combination	121	33.7	29	24.6	202	69.4	< 0.001
Levosimendan	32	8.9	4	3.4	21	7.2	0.137
Dopamine	1	0.3	0	0.0	1	0.3	1.000
Isoprenaline	11	3.1	2	1.7	19	6.5	0.035
Antiarrhythmic	141	39.3	36	30.5	121	41.6	0.111
Transfusion	25/358	7.0	14/118	11.9	89/291	30.6	< 0.001
Fibrinolysis	6/358	1.7	1/118	0.9	6/291	2.1	0.807
Organ replacement therapies, *n* (%)							
Mechanical circulatory support	23/358	6.4	10/118	8.5	107/291	36.8	< 0.001
if yes:							
IABP	15/23	65.2	4/9	44.4	29/107	27.1	0.002
Impella	4/23	17.4	0/9	0.0	22/107	20.6	0.419
VA-ECMO	4/23	17.4	4/9	44.4	76/107	71.0	< 0.001
Renal replacement therapy	30	8.4	10	8.5	79	27.2	< 0.001
Invasive cardiology, *n* (%)							
CAG	168	46.8	56	47.5	173	59.5	0.004
If yes:							0.009
CAG result							
Normal	42	25.0	10	17.9	22	12.7	
1-Mono	26	15.5	12	21.4	41	23.7	
2-Bi	43	25.6	15	26.8	33	19.1	
3-Tri	39	23.2	11	19.6	37	21.4	
Unknown	18	10.7	8	14.3	40	23.1	
Culprit lesion	101/126	80.2	36/46	78.3	119/145	82.1	0.829
Any PCI	84	23.4	29	24.6	104	35.7	0.001
Any PCI (even in a second time)	91	25.4	30	25.4	105	36.1	0.007
Right heart catheterisation	44	12.3	18	15.3	59	20.3	0.020
Pace-maker implantation	18/340	5.3	5/116	4.3	12/276	4.4	0.832
Defibrillator implantation	21/340	6.2	5/116	4.3	11/276	4.0	0.431
Radiofrequency ablation	10/340	2.9	3/116	2.6	4/276	1.5	0.426
Discharge parameters							
LVEF (%), mean +/− SD	31.7	+/− 13.9	32,1	+/− 13.3	41	+/− 14.0	< 0.001
* n*	*207*		*82*		*150*		
LVEF variation*, mean +/− SD	5.5	+/− 11.0	4,5	+/− 11.1	14,7	+/− 17.3	< 0.001
* n*	*205*		*81*		*150*		
Length of stay in ICU/ICCUU (days), median (IQR)	10 (7–16)		11 (6–18)		14 (8–26)		0.002
* n*	*196*		*64*		*180*		
Length of stay in hospital (days), median (IQR)	16 (11–24)		16 (10–28)		20 (13–37)		0.001
* n*	*219*		*78*		*139*		
Discharge mode							
Home	89	29.9	29	28.7	53	21.5	
Rehabilitation	22	7.4	12	11.9	10	4.0	
Transfered (other center/other department)	103	34.6	34	33.7	77	31.2	
Death	83	27.9	24	23.8	107	43.3	
Other	1	0.3	2	2.0	0	0.0	
Registration on transplant waiting list	13/275	4.7	7/112	6.3	20/235	8.5	0.221
Prognosis							
30-day mortality	85	23.7	22	18.6	92	31.6	0.011
30-day MACE	99	27.6	27	22.9	113	38.8	0.001
1-year mortality**	152	42.3	49	41.5	145	49.8	0.114

*CAG* coronary artery angiography, *VA-ECMO* venoarterial-extracorporeal membrane oxygenation, *IABP* intra-aortic balloon pump, *ICU* intensive care unit, *ICCU* intensive cardiac care unit, *LVEF* left ventricular ejection fraction, *MACE* major cardiovascular adverse event defined by death or heart transplantation, or LVAD/BiVAD support, *PCI* percutaneous coronary intervention

^*^At discharge compared with admission

^**^Sachant qu’il y a 3% de perdus de vue à 1 an

Half of CS patients had undergone coronary angiography. A 3-vessel disease was found in about 20% of the cases but only the culprit lesion was revascularized in about 80% without significant differences between groups. There were also no differences regarding right-heart catheterization, pacemaker or defibrillator implantation, or radiofrequency ablation.

### CS prognosis according to ventilatory support

At discharge, LVEF was significantly higher in patients in the MV group (41 vs 32.1 vs 31.7%, *p* < 0.01). Patients in the MV group were hospitalized longer (20 vs 16 days, *p* < 0.01) but without difference in the discharge mode (home, rehabilitation, or care center).

**Fig. 1:**
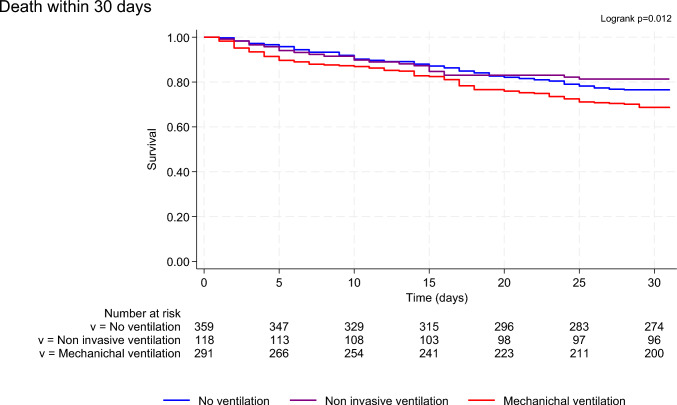
30 day survival according to the maximal level of ventilatory support used during hospitalization

**Fig. 2:**
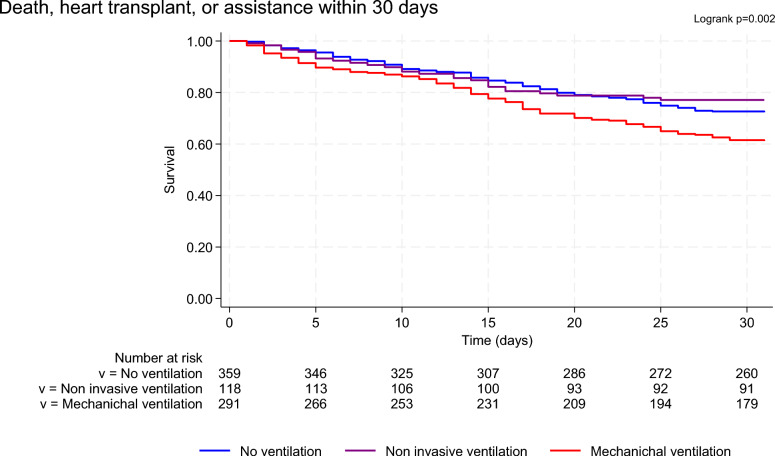
30 day survival free from heart transplantation or LVAD/BiVAD support according to the maximal level of ventilatory support used during hospitalization. BiVAD, biventricular assist device or total artificial heart; LVAD, left ventricular assist device

At 30 days, MV group presented higher mortality (crude HR (MV vs No Ventilation) 1.41 [1.05–1.90], *p* = 0.022 and Fig. 1, log-rank *p* = 0.012) and a higher rate of major adverse events (death, heart transplantation or ventricular assistance) as compared with others groups (crude HR (MV vs No Ventilation) 1.52 [1.16–1.99], *p* = 0.003 and Fig. 2, log-rank *p* = 0.002). At 1 year, the between-groups difference subsists (Supplementary Fig. 1, log-rank *p* = 0.052), especially with a higher mortality for MV patients (crude HR (MV vs No Ventilation) 1.28 [1.02–1.61], *p* = 0.032).

Interestingly after adjustment for known independent predictors of 30-day mortality [5] (age, LVEF < 30%, mechanical circulatory support, RRT, use of norepinephrine and use of diuretics), the between groups difference in 30-day all-cause mortality disappears (Supplementary Table 1).

No difference in all-cause mortality (Crude HR (NIV vs No Ventilation) 0.79 [0.49–1.26], *p* = 0.315) or MAE (Crude HR (NIV vs No Ventilation) 0.83 [0.54–1.27], *p* = 0.399) was found between NIV and NV groups either at 30-day or 1-year (Crude HR (NIV vs No Ventilation) 0.95 [0.69–1.31], *p* = 0.752 for all-cause mortality).

Among MV group, no difference in terms of 30-day mortality was found between patients intubated directly (*n* = 237, 84%) and patients first ventilated by NIV and then intubated (*n* = 44, 16%) (respectively 31.7 and 31.8%, *p* = 0.899) (Supplementary Fig. 2). No difference was found neither between patients intubated directly, patients first ventilated by NIV and then intubated within 24 h (*n* = 28, 10%), or patients first ventilated by NIV and then intubated after 24 h (*n* = 16, 6%) (respectively 31.7, 28.6 and 37.5%, *p* = 0.899) (Supplemental Fig. 3).

### Factors associated with the use of invasive mechanical ventilation

Factors associated with increased use for MV (Table 4) are previous cardiac arrest (OR 5.48, *p* < 0.001), infectious CS trigger (OR 2.55, *p* 0.001), presence of mottling (OR 2.25, *p* < 0.01), and higher lactate at admission (OR 2.13 for the third tertile of lactate, *p* 0.003). On the other hand, older patients (OR 0.97 for 1 year more, *p* < 0.001), non-observant patients (OR 0.17; *p* = 0.001), and those on long-term furosemide were less managed by MV (OR 0.47; *p* = 0.006).

**Table 4:** Factors associated with the use of invasive mechanical ventilation

		Odds ratio	95% CI	*p*
Age (years)*		0.97	0.95–0.98	< 0.001
Trigger: infection		2.55	1.49–4.35	0.001
Trigger: non compliance		0.17	0.05–0.60	0.006
Ongoing furosemide treatment		0.47	0.32–0.68	< 0.001
Cardiac arrest		5.48	2.89–10.39	< 0.001
Mottling		2.25	1.54–3.29	< 0.001
Lactates				
	Tertile 1	1.00	Ref	
	Tertile 2	1.33	0.85–2.08	0.210
	Tertile 3	2.13	1.30–3.48	0.003
	Unknown	0.30	0.12–0.70	0.006

*n* = 657. Hosmer – Lemeshow goodness of fit *p* = 0.327

*CI* confidence interval

^*^For 1 year more

## Discussion

To our knowledge, this study is one of the first studies to provide information about contemporary use of different ventilation modalities and their associated outcomes in a large, unselected cohort of CS patients. First, we reported the use of ventilatory support in 1 on 2 CS patients with NIV alone in 15.4% of the cases and MV in 37.9%. Second, compared to NV and NIV, MV was associated with the worst prognosis at 30 days in terms of all-cause mortality and MAE (mortality, heart transplantation, or LVAD/BiVAD) probably due to a more severe CS presentation. Third and more importantly, NIV was not associated with increased mortality or MAE even after adjustment for severity of disease as compared with patients not ventilated. Fourth, MV was used for more severe and younger patients with mixed shock.

When APE is accompanied with reduced LV systolic function, the application of positive end-expiratory pressure (PEEP) offers several theoretical benefits [21]. PEEP can effectively alleviate congestion by decreasing venous return, increasing transmural pressure, and reducing LV afterload. These mechanisms collectively contribute to the enhancement of oxygenation levels, mitigation of hypercapnia, and alleviation of acidosis. In addition, MV can lessen the burden of breathing workload, leading to a reduction in myocardial oxygen consumption when combined with PEEP.

Data about ventilatory support strategies in CS are scarce. *Hongisto *et al. reported the use of MV in 63% and NIV in 12% of their 219 CS patients from diverse etiologies [14]. The inclusion of patients in different centers (from primary to tertiary) and unit (ICU and ICCU), and the use of a specific definition (FRENSHOCK definition) of CS allowing inclusion of patients from ischemic and non-ischemic etiologies but also patients with less profound shock [5, 20], probably explain lower use of ventilatory support especially MV in our cohort (15.4 and 37.9% of patients with NIV and MV, respectively). Furthermore, patients were categorized according to the maximal level of ventilatory support used during hospitalization in our registry whereas they defined their groups according to the maximum intensity of ventilatory support during the first 24 h of management. As indication, type, and timing of ventilatory support not only depend on shock severity but also on clinicians’ expertise and habits, we assume that the use of the maximum level of support during hospitalization probably better reflects CS severity and evolution.

As previously observed, MV was associated with severe prognosis with higher 30-day mortality and MAE in our cohort as compared with other groups reflecting in part more severe shock [14]. Interestingly, we did not find any difference in terms of mortality or MAE at 30 days between NIV and NV groups. This is a major point of this study since to date, place of NIV in CS management is obvious: at best not recommended and at worst contraindicated by consensus or guidelines [1, 2, 9–11]. The decision to intubate and start invasive MV is often multi-factorial considering respiratory (clinical signs of respiratory failure, oxygenation index), neuropsychological (agitation, consciousness disorders) and hemodynamic parameters (vasopressor dose, lactate level and progress in multiorgan failure or implantation of an acute mechanical support) [21]. To date, however, there is no consensus or guidelines specifying the category of patients most likely to benefit from intubation in circulatory shock states, nor is there guidance on the optimal timing for it, due to the lack of available evidence. Caution is only advised in case of hypotension or right ventricular dysfunction due to possible undesirable effect of PEEP on right ventricular afterload and function. Moreover, due to ventricular interdependence, NIV influences on the RV can ultimately affect left ventricular (LV) performance. A dilated, pressure-overloaded RV can displace the interventricular septum toward the LV, decreasing LV preload and stroke volume. Guidelines do not recommend using NIV in patients presenting with acute coronary syndrome or APE and suffering from shock or low blood pressure, or requiring urgent coronary revascularization. In many studies regarding the use of NIV in APE or acute kidney injury, the presence of low blood pressure, need for vasoactive medications or shock have been considered as exclusion criteria or as criteria for intubation [15–17].

NIV presents several advantages as compared with MV. NIV allows patients to communicate, eat, move at least to some extent, and breathe spontaneously. By avoiding endotracheal intubation and invasive MV, the risks of nosocomial infections, ventilator-associated pneumonia, and injuries related to the intubation procedure itself are diminished [22]. Moreover, the use of profound sedation with loss of vasomotor tone can be avoided, this might be especially beneficial in patients presenting with symptoms of shock, in whom the sedatives may further increase hypotension [22, 23].

Our study suggests that using NIV is safe in selected patients with less profound shock CS and no other MV indication (mixed shock, post-cardiac arrest management). But special attention should be paid to CS patients under NIV support due to the risk of worsening hypotension and state of consciousness. Patients should be managed and monitored closely and promptly intubated without improvement or in case of degradation (respiratory and/or hemodynamic) under NIV support.

Future dedicated studies should prospectively investigate this major topic, but none are currently reported on clinicaltrial.gov.

## Limitations

There are some limitations to be acknowledged. First, data from patients who died before informed consent was obtained were not collected and recorded in the database because of administrative regulations. Thus, it cannot be excluded that the most severe patients i.e., with several comorbidities, frailty, or multiple end-stage organ failure could not have been admitted in ICU/ICCU for futility or have been deceased before inclusion. This could be a source of bias resulting in an underestimation of mortality. Second, the choice of ventilation strategy was not protocolized and was left at the discretion of the physician in charge. In addition, the specific reasons for intubation and MV (for example, potential confusion / neurologic dysfunction from CS, hemodynamic instability, aMCS location) are unfortunately not available in our data set because our register does not have been designed for this. However, as our study was carried out in line with the state of the art, is multicentric and included ICCU and ICU patients, we believe it reflects the usual indications for the use of NIV and MV, mainly based on “common sense”, hemodynamic, respiratory and neurologic parameters [21]. Timing and escalation of ventilation strategies were based on standard clinical practice and progressive management (where possible), which involved starting with NIV, followed by therapeutic escalation with IMV as recently recommended by expert advice [25]. However, the study reflects real-life practice in university and non-university, public and private hospitals in France through a large nationwide collection of CS patients. Third, a global severity score such as the SOFA score, the SAPS II, or the Charlson comorbidity index would have been useful for comparing the initial severity of different groups of patients. But these scores were not recorded, and we were not able to use the SCAI SHOCK Stage Classification given that it was not yet available at the time of our study.

## Conclusion

Due to the lack of available data, levels of evidence to guide ventilatory support strategies in CS patients are low to date. In this large prospective nationwide registry of unselected CS, we report that NIV can be safely used for respiratory failure management in properly selected CS patients in clinical practice. Nevertheless, given the increasing use of ventilation in cardiogenic shock, futures dedicated studies appear necessary to address this issue and confirm our findings.

## Supplementary Information

Below is the link to the electronic supplementary material.

Supplementary file1 (DOCX 64 KB)

## Data Availability

Data are available from the corresponding author upon reasonable request.

## Abbreviations

CI: Cardiac index
CRP: C-reactive protein
CRT: Capillary refill time
CS: Cardiogenic shock
ESC: European Society of Cardiology
ESICM: European Society of Intensive Care Medicine
eGFR: Estimated glomerular filtration rate
IABP: Intra-aortic balloon pump
ICCU: Intensive cardiac care unit
ICU: Intensive care unit
LVEF: Left ventricular ejection fraction
MAE: Major adverse events
MAP: Mean arterial pressure
MI: Myocardial infarction
MCS: Mechanical circulatory support
MV: Mechanical ventilation
NIV: Noninvasive ventilation
NV: No ventilation
PCI: Percutaneous coronary intervention
RRT: Renal replacement therapy
SAP: Systolic arterial pressure
VA-ECMO: Venoarterial-extracorporeal membrane oxygenation

## References

[1] Van Diepen S, Katz JN, Albert NM, Henry TD, Jacobs AK, Kapur NK (2017) Contemporary management of cardiogenic shock: a scientific statement from the american heart association. Circulation 136(16):e232–e268 28923988 10.1161/CIR.0000000000000525

[2] Chioncel O, Parissis J, Mebazaa A, Thiele H, Desch S, Bauersachs J (2020) Epidemiology, pathophysiology and contemporary management of cardiogenic shock- a position statement from the heart failure association of the european society of cardiology. Eur J Heart Fail 22(8):1315–1341 32469155 10.1002/ejhf.1922

[3] Zeymer U, Vogt A, Zahn R, Weber MA, Tebbe U, Gottwik M (2004) Predictors of in-hospital mortality in 1333 patients with acute myocardial infarction complicated by cardiogenic shock treated with primary percutaneous coronary intervention (PCI): results of the primary PCI registry of the Arbeitsgemeinschaft Leitende Kardiologische Krankenhausarzte (ALKK). Eur Heart J 25(4):322–328 14984921 10.1016/j.ehj.2003.12.008

[4] Jeger RV, Radovanovic D, Hunziker PR, Pfisterer ME, Stauffer JC, Erne P (2008) Ten-year trends in the incidence and treatment of cardiogenic shock. Ann Intern Med 149:618–626 18981487 10.7326/0003-4819-149-9-200811040-00005

[5] Delmas C, Roubille F, Lamblin N, Bonello L, Leurent G, Levy B (2022) Baseline characteristics, management, and predictors of early mortality in cardiogenic shock: insights from the FRENSHOCK registry. ESC Heart Fail 9(1):408–419 34973047 10.1002/ehf2.13734 PMC8788015

[6] Aissaoui N, Puymirat E, Delmas C, Ortuno S, Durand S, Bataille V (2020) Trends in cardiogenic shock complicating acute myocardial infarction. Eur J Heart Failure 22(4):664–672 10.1002/ejhf.1750 32078218

[7] Aissaoui N, Puymirat E, Tabone X, Charbonnier B, Schiele F, Lefevre T (2012) Improved outcome of cardiogenic shock at the acute stage of myocardial infarction: a report from the USIK 1995, USIC 2000, and FAST-MI French nationwide registries. Eur Heart J 33:2535–2543 22927559 10.1093/eurheartj/ehs264

[8] Vallabhajosyula S, Kashani K, Dunlay S, Vallabhajosyula S, Vallabhajosuyla S, Sundaragiri P (2019) Acute respiratory failure and mechanical ventilation in cardiogenic shock complicating acute myocardial infarction in the USA, 2000–2014. Ann Intensive Care 9(1):96 31463598 10.1186/s13613-019-0571-2 PMC6713772

[9] Werdan K, Russ M, Buerke M, Delle-Karth G, Geppert A, Schondube FA (2012) Cardiogenic shock due to myocardial infarction: diagnosis, monitoring and treatment: a german-austrian S3 guideline. Dtsch Arztebl Int 109:343–351 22675405 10.3238/arztebl.2012.0343 PMC3364528

[10] Keenan S, Sinuff T, Burns K, Muscedere J, Kutsogiannis J, Sangeeta M (2011) Clinical practice guidelines for the use of noninvasive positive-pressure ventilation and noninvasive continuous positive airway pressure in the acute care setting. CMAJ 183(3):E195-214 21324867 10.1503/cmaj.100071 PMC3042478

[11] Masip J, Peacock F, Price S, Cullen L, Martin-Sanchez J, Seferovic P (2018) Indications and practical approach to non-invasive ventilation in acute heart failure. Eur Heart J 39(1):17–25 29186485 10.1093/eurheartj/ehx580 PMC6251669

[12] Zyemer U, Bueno H, Granger C, Hochman J, Huber K, Lettino M (2020) Acute cardiovascular care association position statement for the diagnosis and treatment of patients with acute myocardial infarction complicated by cardiogenic shock: a document of the acute cardiovascular care association of the european society of cardiology. Eur Heart J Acute Cardiovasc Care 9(2):183–197 32114774 10.1177/2048872619894254

[13] Mac Donagh TA, Metra M, Adamo M, Gardner R, Baumbach A, Böhm M (2022) 2021 ESC Guidelines for the diagnosis and treatment of acute and chronic heart failure: developed by the task force for the diagnosis and treatment of acute and chronic heart failure of the European Society of Cardiology (ESC). With the special contribution of the heart failure association (HFA) of the ESC. Eur J Heart Fail 24(1):4–131 35083827 10.1002/ejhf.2333

[14] Hongisto M, Lassus J, Tarvasmaki T, Sionis A, Tolppanen H, Greve LM (2017) Use of noninvasive and invasive mechanical ventilation in cardiogenic shock: A prospective multicenter study. Int J Cardiol 1(230):191–197 10.1016/j.ijcard.2016.12.175 28043661

[15] Masip J, Betbese AJ, Paez J, Vecilla F, Canizares F, Padro J (2000) Non-invasive pressure support ventilation versus conventional oxygen therapy in acute cardiogenic pulmonary oedema: a randomised trial. Lancet 356(9248):2126–2132 11191538 10.1016/s0140-6736(00)03492-9

[16] Park M, Sangean MC, Volpe MS, Feltrim M, Nozawa E, Leite P (2004) Randomized, prospective trial of oxygen, continuous positive airway pressure, and bilevel positive airway pressure by face mask in acute cardiogenic pulmonary edema. Crit Care Med 32(12):2407–2415 15599144 10.1097/01.ccm.0000147770.20400.10

[17] Vital FM, Ladeira MT, Atallah AN (2013) Non-invasive positive pressure ventilation (CPAP or bilevel NPPV) for cardiogenic pulmonary oedema. Cochrane Database Syst Rev. 10.1002/14651858.CD005351.pub3 23728654 10.1002/14651858.CD005351.pub3

[18] Gimenez M, Miller P, Alviar C, Diepen S, Granger C, Montalescot G (2020) Outcomes associated with respiratory failure for patients with cardiogenic shock and acute myocardial infarction: a substudy of the CULPRIT-SHOCK trial. J Clin Med 9(3):860 32245139 10.3390/jcm9030860 PMC7141492

[19] Van Diepen S, Hochman J, Stebbins A, Alviar C, Alexander J, Lopes R (2020) Association between delays in mechanical ventilation initiation and mortality in patients with refractory cardiogenic shock. JAMA Cardiol 5(8):965–967 32432650 10.1001/jamacardio.2020.1274 PMC7240630

[20] Delmas C, Puymirat E, Leurent G, Elbaz M, Manzo-Silberman S, Bonello L, Gerbaud E, Bataille V, Levy B, Lamblin N et al (2019) Design and preliminary results of FRENSHOCK 2016: a prospective nationwide multicentre registry on cardiogenic shock. Arch Cardiovasc Dis 112(5):343–353 30982720 10.1016/j.acvd.2019.02.001

[21] Darreau C, Martino F, Saint-Martin M et al (2020) Use, timing and factors associated with tracheal intubation in septic shock: a prospective multicentric observational study. Ann Intensive Care 10:62 32449053 10.1186/s13613-020-00668-6 PMC7245631

[22] Alviar CL, Miller PE, McAreavey D, Katz JN, Lee B, Moriyama B et al (2018) Positive pressure ventilation in the cardiac intensive care unit. J Am Coll Cardiol 72:1532–1553 30236315 10.1016/j.jacc.2018.06.074 PMC11032173

[23] Girou E, Schortgen F, Delclaux C, Brun-Buisson C, Blot F, Lefort Y (2000) Association of noninvasive ventilation with nosocomial infections and survival in critically ill patients. JAMA 284(18):2361–2367 11066187 10.1001/jama.284.18.2361

[24] Mort TC (2007) Complications of emergency tracheal intubation: hemodynamic alterations–part I. J Intensive Care Med 22(3):157–165 17562739 10.1177/0885066607299525

[25] Alviar CL, Rico-Mesa JS, Morrow DA et al (2020) Positive pressure ventilation in cardiogenic shock: review of the evidence and practical advice for patients with mechanical circulatory support. Can J Cardiol 36:300–312 32036870 10.1016/j.cjca.2019.11.038

