# Supplementary material for: Ventilation strategies in cardiogenic shock: insights from the FRENSHOCK observational registry
Source: Clin Res Cardiol. 2024 Oct 23;114(10):1311–23. doi: 10.1007/s00392-024-02551-x (PMC12460561; doi:10.1007/s00392-024-02551-x)
Supplement: Supplementary file 1 — Supplementary file1 (DOCX 64 KB) [file 392_2024_2551_MOESM1_ESM.docx]

**Supplementary data**

**"Ventilation Strategies in Cardiogenic Shock: Insights from the Frenshock Observational Registry - Volle.K et al**

- **Supplemental Table 1: Effect of maximal ventilatory support used during hospitalization on 30-day all-cause mortality after adjustment for known independent predictors of 30-day mortality.**
- **Supplemental Figure 1: 1-year survival according to the maximal level of ventilatory support used during hospitalization.**
- **Supplemental Figure 2: 30-day survival according to strategy of ventilatory support used during hospitalization in patients ventilated (patients directly intubated vs patients first ventilated by non-invasive ventilation and then intubated).**
- **Supplemental Figure 3: 30-day survival according to strategy and timing of ventilatory support used during hospitalization in patients ventilated.**

**Supplemental Table 1: Effect of maximal ventilatory support used during hospitalization on 30-day all-cause mortality after adjustment for known independent predictors of 30-day mortality.**

|  | **Crude HR** | **95% CI** | **p** | **Adjusted HR *** | **95% CI** | **p** |
| --- | --- | --- | --- | --- | --- | --- |
| No ventilation | 1.00 | (reference) |  | 1.00 | (reference) |  |
| Non invasive ventilation | 0.79 | 0.48 - 1.28 | 0,336 | 0.75 | 0.46 – 1.23 | 0.257 |
| Mechanical ventilation | 1.45 | 1.06 - 1.99 | 0,022 | 0.97 | 0.66 – 1.41 | 0.861 |
| * adjusted for independent predictors of 30d mortality : age, LVEF<30%, mechanical circulatory support, renal replacement therapy, use of norepinephrine and use of diuretics (Delmas.C et al, ESC Heart Fail 2022) | | | | | | |

**Supplemental Figure 1: 1-year survival according to the maximal level of ventilatory support used during hospitalization**

Crude HR (NIV vs No Ventilation) 0.95 [0.69 - 1.31] p=0.752

Crude HR (MV vs No Ventilation) 1.28 [1.02 - 1.61] p=0.032

**Supplemental Figure 2: 30-day survival according to strategy of ventilatory support used during hospitalization in patients ventilated (patients directly intubated vs patients first ventilated by non-invasive ventilation and then intubated)**

Crude HR (later vs immediately) 1.04 [0.59 - 1.84] p=0.894

**Supplemental Figure 3: 30-day survival according to strategy and timing of ventilatory support used during hospitalization in patients ventilated.**

MV, mechanical ventilation ; NIV, non invasive mechanical ventilation

Crude HR (NIV same day vs MV immediately) 0.95 [0.46 - 1.96] p=0.883

Crude HR (NIV then MV later vs MV immediately) 1.19 [1.02 - 1.61] p=0.032
